# Complete genome sequence of a novel fish papillomavirus detected in farmed wels catfish (*Silurus glanis*)

**DOI:** 10.1007/s00705-021-05123-w

**Published:** 2021-06-11

**Authors:** András Surján, Eszter Fónagy, Edit Eszterbauer, Balázs Harrach, Andor Doszpoly

**Affiliations:** Institute for Veterinary Medical Research, Centre for Agricultural Research, ELKH, 1143 Budapest, Hungária krt. 21., P.O. Box 18, 1581 Budapest, Hungary

## Abstract

A novel papillomavirus (PV) was detected in farmed wels catfish (*Silurus glanis*) in Hungary showing clinical signs resembling those of wels catfish herpesvirus disease. The whole genome of Silurus glanis papillomavirus 1 (SgPV1) was identified using next-generation sequencing. The 5,612-bp complete genome contains four predicted protein coding regions (E1, E2, L1, and L2), which seem to have homologues in every PV genome sequenced to date. Five complete fish PV genome sequences are available in the GenBank database. Their genomes range between 5,748 and 6,086 bp and contain the minimal PV backbone genes E1, E2, L2, and L1, unlike PVs of higher vertebrates, which have larger genomes (6.8-8.6 kbp) and additional (onco)genes. Considering the current species demarcation criteria for the family *Papillomaviridae*, the establishment of a novel species named "*Nunpapillomavirus siluri*" is proposed for the SgPV1 in a novel genus, "*Nunpapillomavirus*"*,* in the subfamily *Secondpapillomavirinae*.

**Supplementary Information:**

The online version contains supplementary material available at 10.1007/s00705-021-05123-w.

Papillomaviruses (PVs) are small (55 nm in diameter), icosahedral, non-enveloped viruses with circular, double-stranded DNA. Their genomes range between 5.7 and 8.6 kilobases (kb) [10]. PVs have been reported in members of almost every vertebrate class: Mammalia, Aves, Reptilia, and Actinopterygii [5, 8]. Pathogenic PVs usually cause benign epithelial tumors known as papillomas. All known PVs belong to the family *Papillomaviridae*, and their classification is based on pairwise nucleotide sequence identity in the major capsid protein (L1) gene. The family consists of two subfamilies: *First*- and *Secondpapillomavirinae*, the latter of which includes one genus (*Alefpapillomavirus*) with a single species, *Alefpapillomavirus 1* (Sparus aurata PV1). In the subfamily *Firstpapillomavirinae*, there are more than 50 genera with over 130 species recognized by the International Committee on Taxonomy of Viruses (ICTV), with all of their members originating from higher vertebrates [10].

The first identified fish PV, Sparus aurata PV1, was reported in 2016 in gilt-head seabream (*Sparus aurata*) showing typical signs of lymphocystivirus disease. The whole genome of the associated lymphocystivirus (LCDV) was identified by next-generation sequencing, and additionally, the first fish PV and a novel polyomavirus (PyV) were discovered [5]. Four additional complete genome sequences of fish PVs are now available in the GenBank database. They were detected in a mass metagenomic study ranging from nematodes to human tissue samples enriched for circular DNA viruses [9]. The latter four viruses are still considered unclassified members of the family *Papillomaviridae*. In the present study, the complete genome of the sixth fish PV (i.e., that of Silurus glanis PV1; SgPV1) was sequenced and analysed.

Specimens from diseased wels catfish (2+ years of age) showing multiple papilloma-like epidermal hyperplasia on the skin were collected at a fish farm in Hungary in 2017 (Fig. 1). The clinical signs were similar to those described previously for herpesvirus infection in wels catfish in Hungary [2]. Samples from the skin lesions were homogenized, and cell debris removed by centrifugation at 5,000 × *g*. Subsequently, the virions were concentrated by ultracentrifugation at 139,000 × *g*, and the viral DNA was extracted by the phenol/chloroform extraction method. A short-insert DNA library was prepared using a Nextera XT DNA Library Preparation Kit (Illumina, USA) and sequenced using an Illumina HiSeq 2000 platform (Illumina, USA). A total of 4,597,506 paired-end reads were generated, with an average length of 102 bp. CLC Genomics Workbench 12.0 (CLC bio, Denmark) was used for genome assembly and annotation. The deduced amino acid (aa) sequences of three genes (E1, E2, and L1) were concatenated and analysed phylogenetically. For tree inference, a multiple alignment of the concatenates was made using MAFFT v7 [4] with default parameters, and the alignment was edited manually. The maximum-likelihood (PhyML) program of the TOPALi v2.5 program package [6] was used with the RTRev amino acid substitution model (1,000 samplings). The phylogenetic tree was visualized using MEGA X [3]. Pairwise sequence identity analysis was performed on the L1 gene of the fish PVs using SDT 1.2 [7]. To test whether the virus has a circular genome, an inverse PCR was developed for amplifying almost the entire genome using Phusion High-Fidelity DNA Polymerase (Thermo Fisher Scientific). A specific PCR assay targeting the E2 gene of the novel PV was also designed for screening five other fish specimens from the same fish farm showing similar clinical signs. The assay was performed using DreamTaq Hot Start PCR Master Mix (Thermo Fisher Scientific). The sequences of the oligonucleotides and the PCR conditions are shown in Supplementary Tables S1 and S2, respectively. PCR products extracted from an agarose gel were purified using a NucleoSpin Extract II Kit (Macherey-Nagel GmbH & Co). Sanger DNA sequencing was performed using a BigDye Terminator v3.1 Cycle Sequencing Kit (Life Technologies, Thermo Fisher).

**Fig. 1:**
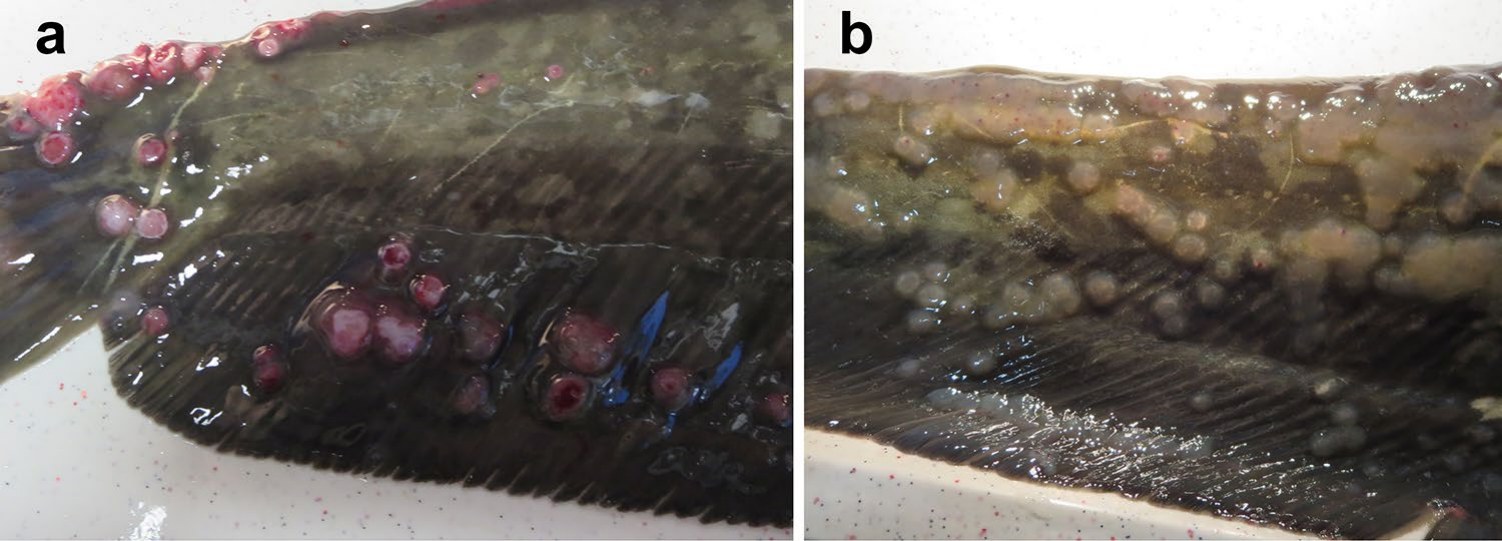
(a and b) Multiple papillomas on the skin of wels catfish. The clinical signs corresponded to those caused by wels catfish herpesvirus

During an attempt to analyze the virome of wels catfish tissues from a fish specimen affected by skin lesions usually attributed to infections by herpes-like viruses, the resulting dataset contained a fully assembled sequence genome of a novel PV. From the putative alloherpesvirus that had been assumed to be the cause of the diseased skin condition, only relatively short sequences were obtained with low coverage (data not shown).

The SgPV1-specific diagnostic PCR targeting the E2 gene gave positive results (with 100% nucleotide sequence identity) in three out of the five additionally tested catfish. Thus, of the six herpesvirus-infected wels catfish (the same specimens studied by Tarján et al., unpublished), four were infected with SgPV1 as well. Coinfection of fish PVs with other DNA viruses in gilt-head seabream was reported previously; out of 10 LCDV-infected seabreams, eight were positive for PV and eight for PyV as well (six fish were infected by both viruses) [5].

Our study revealed that the size of the SgPV1 genome was 5,612 bp (average read depth, 283), and it contained four predicted protein-coding regions (E1, E2, L1, and L2) (Fig. 2a). The genome sequence was deposited in the GenBank database under the accession no. MN515404. The inverse PCR resulted in an amplicon of 5,352 bp, demonstrating that SgPV1 has a circular genome and that it is not an endogenous viral element in the genome of the host. The genome sequence of the novel virus is similar to those of previously described fish PVs. These genomes seem to be unique, as fish PVs are the only papillomaviruses that contain the minimal PV backbone genes (E1-E2-L2-L1), lacking any of the oncogenes (E5, E6, and E7) reported previously by Willemsen and Bravo [11].

**Fig. 2:**
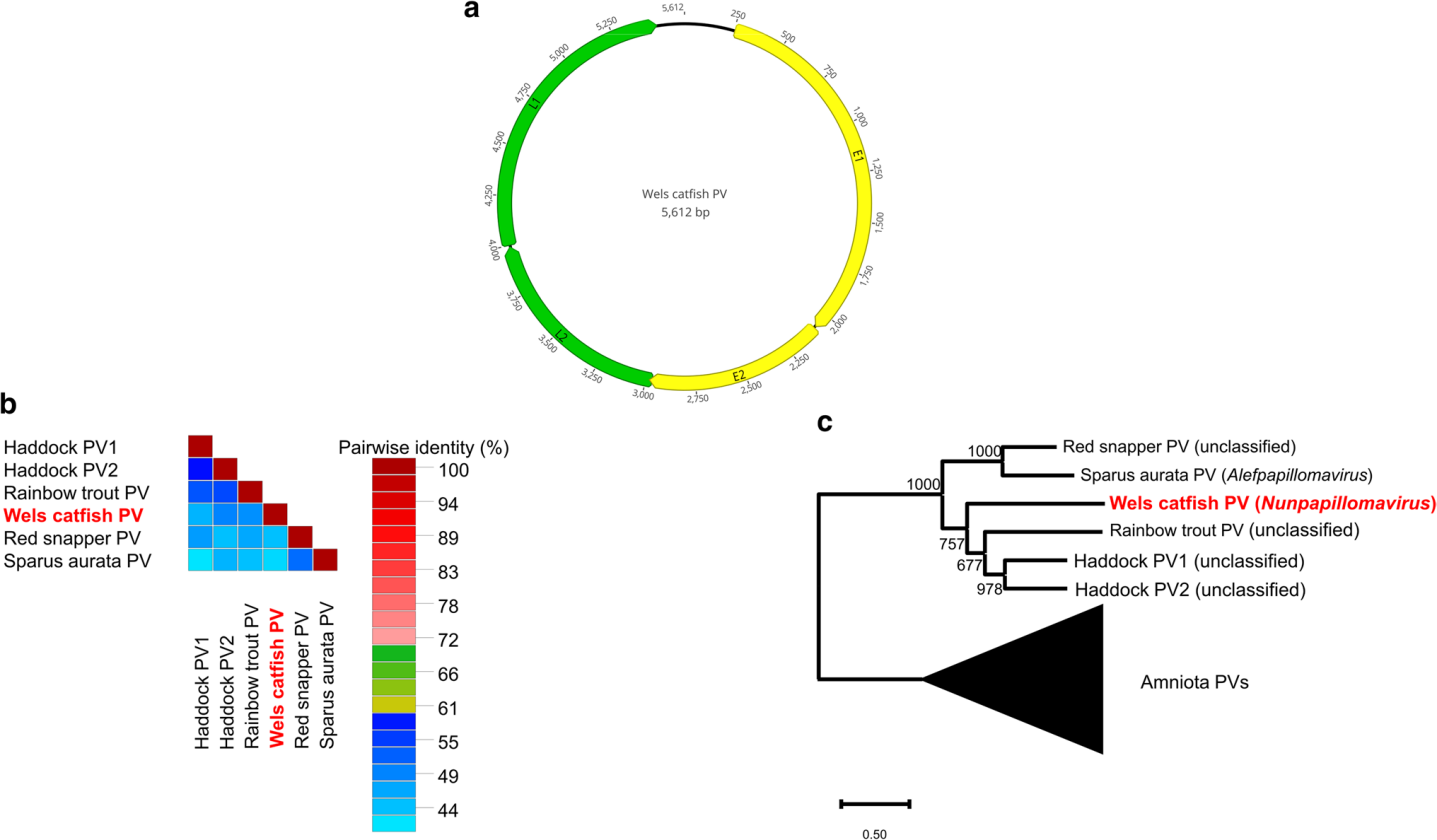
(a) Genome organization of the wels catfish papillomavirus. Green boxes indicate the late genes; yellow boxes indicate the early genes; (b) Pairwise sequence comparisons of L1 core gene amino acid (aa) sequences of fish papillomaviruses (PVs). Pairwise sequence identity percentages are represented using different colours. Cutoff values: 60% and 70%. Blue squares represent sequence identity below 60%; green squares, 60-70%; red, ≥70%. (c) Phylogenetic analysis of PVs based on maximum-likelihood analysis of the concatenated aa sequences of the E1, E2, and L1 genes (1,191 aa). The tree was rooted at the midpoint. The subtree of all amniota (reptiles, birds, and mammals) PVs was compressed

A pairwise alignment of L1 gene sequence showed less than 60% identity to that of any other fish PV (Fig. 2b). The highest identity was to haddock PV2 (48%). Phylogenetic analysis showed that the novel PV unambiguously clusters with members of the subfamily *Secondpapillomavirinae* and showed a clear separation of the novel PV from the previously described viruses (Fig. 2c). Based on these findings and considering the species demarcation criteria for the family *Papillomaviridae* (viruses with >70% identity in the L1 sequence belong to the same species; those >60% identity belong to the same genus), we propose the establishment of a novel species for SgPV1 named "*Nunpapillomavirus siluri*" in a novel genus, "*Nunpapillomavirus*"*,* in the subfamily *Secondpapillomavirinae*. In this subfamily, the first and so far only genus was named after the first letter (Alef) of the Semitic alphabet (Phoenician, Aramaic, Arabic, etc.), while Nun is the fourteenth letter of this alphabet, meaning snake or fish. The proposed species name would follow the recently accepted ICTV policy for the obligatory Latinized or Latin binomial species naming to be applied for the genus name of the host [1].

Regarding the pathology of SgPV1, further studies are needed to ascertain whether SgPV1 by itself can cause any clinical signs or disease, and whether it is always found in coinfection with wels catfish herpesvirus.

## Supplementary Information

Below is the link to the electronic supplementary material.

**Supplementary Table S1** Oligonucleotide primers used for PCR and Sanger DNA sequencing in this study (DOCX 13 KB)

**Supplementary Table S2** PCR conditions for the SgPV1-specific PCR assays (DOCX 13 KB)

## Data Availability

The genome sequence was deposited in the GenBank database under the accession no. MN515404.
