# Supplementary material for: Complete genome sequence of a novel fish papillomavirus detected in farmed wels catfish (Silurus glanis)
Source: Arch Virol. 2021 Jun 11;166(9):2603–6. doi: 10.1007/s00705-021-05123-w (PMC8321979; doi:10.1007/s00705-021-05123-w)
Supplement: Supplementary file 1 — Supplementary Table S1 Oligonucleotide primers used for PCR and Sanger DNA sequencing in this study (DOCX 13 KB) [file 705_2021_5123_MOESM1_ESM.docx]

**Supplementary table 1**

| **target** | **oligonucleotides** | **product size (bp)** |
| --- | --- | --- |
| complete genome by inverse PCR | SgPV1_full_fo: 5’-CAC TGG TGG TCT ATA CTT CCT CTA T-3’  SgPV1_full_re: 5’-CCT GTT GTT GTC TGT CCA TAA TGT A-3’ | 5352 |
| E2 gene | SgPV1_E2_fo: 5’-CAG AAG CAG CAG ATC CAC CA-3’  SgPV1_E2_re: 5’-GCT TCA GCG TAG TGG TCT GT-3’ | 218 |
