# Supplementary material for: Complete genome sequence of a novel fish papillomavirus detected in farmed wels catfish (Silurus glanis)
Source: Arch Virol. 2021 Jun 11;166(9):2603–6. doi: 10.1007/s00705-021-05123-w (PMC8321979; doi:10.1007/s00705-021-05123-w)
Supplement: Supplementary file 2 — Supplementary Table S2 PCR conditions for the SgPV1-specific PCR assays (DOCX 13 KB) [file 705_2021_5123_MOESM2_ESM.docx]

**Supplementary table 2**

| PCR steps | **temperature (Phusion/DreamTaq)** | **duration (Phusion/DreamTaq)** | **No. of cycles** | | |
| --- | --- | --- | --- | --- | --- |
| initial denaturation | 98/95°C | 3/3 min | | 1 |  |
| denaturation | 98/95°C | 10/30 sec | | 45 |  |
| annealing | 58/65°C | 30/30 sec | | 45 |  |
| elongation | 72/72°C | 3/0.5 min | | 45 |  |
| final extension | 72/72°C | 5/5 min | | 1 |  |
